# A Case of *SF3B1*-Positive Myelodysplastic/Myeloproliferative Neoplasm with Ring Sideroblasts and Thrombocytosis

**DOI:** 10.4274/tjh.galenos.2018.2018.0267

**Published:** 2019-02-07

**Authors:** Alejandro Lazo-Langner, Bekim Sadikovic

**Affiliations:** 1 Western University, Schulich School of Medicine, Department of Medicine, London, Ontario, Canada; 2 Western University, Schulich School of Medicine, Department of Epidemiology and Biostatistics, London, Ontario, Canada; 3 Western University, Schulich School of Medicine, Department of Pathology and Laboratory Medicine, London, Ontario, Canada

**Keywords:** Myelodysplasia, Ring sideroblasts, Splicing factor 3b subunit 1 (SF3B1)

A 77-year-old woman, previously maintained on phlebotomies that had been discontinued 3 years before for a purported diagnosis of iron overload, was assessed for normocytic normochromic anemia. Her blood count showed hemoglobin of 90 g/L (normal: 115-160), mean corpuscular volume of 93.2 fL (normal: 79-97), erythrocyte distribution width of 28.1% (normal: 12%-15%), and platelets of 422x109/L (normal: 150-400). Iron studies showed elevated ferritin (491 µg/L; normal: 13-150), total iron of 14 µmol/L (normal: 7-26), transferrin saturation of 32% (normal: 11%-56%), and unsaturated iron binding capacity of 30 µmol/L (normal: 19.7-66.2). The vitamin B6 level was low (<10 nmol/L; normal: 20-96). HFE C282Y, H63D, and JAK2 V617F mutations were negative. The peripheral blood smear showed marked anisopoikilocytosis (Figure 1A; Wright’s stain, 40x). A bone marrow aspirate and biopsy showed hypercellular marrow (70%-80%) with moderate dyserythropoiesis, minimal dysplastic changes in other lineages, and increased ring sideroblasts (Figure 1B; Perls’ stain, 100x), consistent with a myelodysplastic/myeloproliferative neoplasm with ring sideroblasts and thrombocytosis (MDS/MPN-RS-T; WHO 2016). The karyotype was normal. Next-generation sequencing studies reported the presence of an *SF3B1*:c1986C>A, p.(His662Gln) mutation (Figure 1C) with a variant allele frequency of 40.5%. *SF3B1* mutations result in the disruption of mitochondrial iron metabolism and define a distinct subgroup of patients with myelodysplasia with a better prognosis than other subtypes.

## Figures and Tables

**Figure 1:**
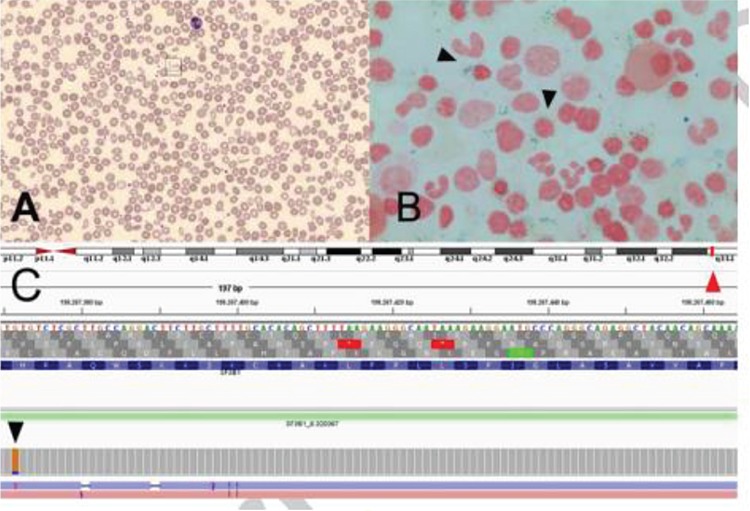
A) Peripheral blood smear (Wright’s stain, 40^x^) showing marked anisopoikilocytosis. B) Bone marrow aspirate (Perls’ stain, 100^x^) showing increased ring sideroblasts (arrowheads). C) Next-generation sequencing pileup plot showing sequencing results for location 2q33.1 (red arrowhead) indicating the presence of an SF3B1:c1986C>A mutation (black arrowhead).

